# The role of exopolymeric substances in the bioaccumulation and toxicity of Ag nanoparticles to algae

**DOI:** 10.1038/srep32998

**Published:** 2016-09-12

**Authors:** Kaijun Zhou, Yi Hu, Luqing Zhang, Kun Yang, Daohui Lin

**Affiliations:** 1 Department of Environmental Science, Zhejiang University, Hangzhou 310058, China; 2 Zhejiang Provincial Key Laboratory of Organic Pollution Process and Control, Zhejiang University, Hangzhou 310058, China

## Abstract

Exopolymeric substances (EPS) have an important role in bioaccumulation and toxicity of nanoparticles (NPs) to algae, which warrants specific studies. The interaction of EPS with citrate and polyvinyl pyrrolidone (PVP) coated AgNPs (C-AgNPs and P-AgNPs, respectively) and its roles in bioaccumulation and toxicity of the AgNPs to *Chlorella pyrenoidosa* were investigated. The amino and aromatic carboxylic groups in the EPS were involved in the EPS-AgNP interactions. Compared with Ag^+^, C-AgNPs had comparable total bioaccumulation but greater absorption by intact algae with EPS; P-AgNPs had the smallest total bioaccumulation and were mainly adsorbed on algal surfaces. With EPS removed, the total bioaccumulations and surface adsorptions for the three Ag species decreased but the cell internalizations increased; the 96 h half growth inhibition concentrations decreased, indicating EPS alleviated the algal toxicity of Ag. The cell-internalized but not the adsorbed AgNPs could contribute to the nanotoxicity. The EPS could bind both AgNPs and Ag^+^, and thus inhibited the cell internalization and the nanotoxicity. However, the EPS-bound Ag on the cell surfaces would migrate along with the algae and be biologically amplified in the aquatic food chains, presenting ecological risks. These results are helpful for understanding the fate and ecological effects of NPs.

Silver nanoparticles (AgNPs) are the most commercialized nanomaterial, having found versatile applications in diverse products such as bactericides, fungicides, household appliances, cleaners, clothing, cutlery, toys, and medical equipments^1^,^2^,^3^. As a result of their wide applications, a considerable fraction of AgNPs can find their way into aquatic ecosystems, be bioaccumulated^4^,^5^,^6^, cause toxic effect mainly by releasing Ag^+^ and inducing ROS generation^7^,^8^,^9^, and potentially threaten plankton like algae^10^,^11^. Algae, as the primary producer and the basis of aquatic food chains, are often used in biological toxicity assays. They can secrete high-molecular polymers, named extracellular polymeric substances (EPS), which form a protective layer outside the cells and protect algae from external interferences. EPS are complex mixtures composed of proteins, polysaccharides, fats, nucleic acids, and inorganic substances, where proteins and polysaccharides are the main contents and account for 70–80% of the total organic carbon (TOC) content of EPS^12^. EPS contain aromatic and aliphatic monomers in their protein fractions, hydrophobic chains in the polysaccharide parts, and plenty of polar functional groups^13^,^14^. EPS can thus have a potential of interaction with various NPs and alter their bioavailability and toxicity, and it is necessary to iterate the potential role of EPS in the ecological impacts of NPs.

Some researchers have studied the effect of EPS on nanotoxicity. It was found that in the presence of NPs, microbes secreted larger amounts of EPS and exhibited a higher tolerance to AgNPs^15^ and Cu-doped TiO_2_^16^. Su *et al*.^17^ removed EPS from *E. coli* cells and found the bactericidal activity of Ag-doped multi-walled carbon nanotubes increased. Zhang *et al*.^18^ examined the toxicity of CdSe quantum dots to *Thalassiosira pseudonana* under different nutrition conditions and found that in the absence of N elements, the extracellular protein content decreased, the dissolved free Cd^2+^ increased, and correspondingly the toxicity of the CdSe quantum dots increased. To date, it is recognized that EPS can alleviate the microbial toxicity of NPs, but the mechanism was generally simply hypothesized to be the inhibition of bioaccumulation of NPs and/or NPs-released toxic ions. The specific EPS-NP interaction and its roles in the bioaccumulation and toxicity of NPs to microbes merit more experimental studies.

Algal accumulation is an important process of NPs’ toxicity and migration in the aquatic ecosystem. NPs can be surface adsorbed and internalized by algae^19^,^20^,^21^. The metal internalization flux is a good indicator of metal toxicity, which may be also applicative for metal NPs^22^,^23^. The surface absorbed NPs, accounting for a large proportion of the total bioaccumulation, can also migrate in aquatic food chains along with the algae and show potential ecological risks. Therefore, the differentiation between the surface adsorption and internalization of metal-based NPs by algae is extremely relevant and necessary when studying the biological effects of metal-based NPs. EPS, as the first barrier of algae to NPs, are bound to play an important role in the bio-nano interaction. But few researches have studied the role of EPS from the specific EPS-NP interaction, much less distinguishing the cell surface adsorption and internalization of NPs.

In this study, citrate and polyvinyl pyrrolidone (PVP) stabilized AgNP suspensions (C-AgNPs and P-AgNPs, respectively) were prepared, and their bioaccumulation and toxicity to *Chlorella pyrenoidosa* with and without EPS were specifically examined. The interactions between the extracted algal EPS and the AgNPs were investigated. The effects of C-AgNPs, P-AgNPs, and Ag^+^ (from AgNO_3_) were compared. The roles of EPS in the bioaccumulation and toxicity of AgNPs were addressed. The results will increase our knowledge on the ecological effect of NPs.

## Results and Discussion

### Characteristics of AgNPs

The synthesized C-AgNPs and P-AgNPs were spherical as shown in Fig. S1 in the Supplementary Information (SI), with the measured particle sizes (n > 100) being 19.0 ± 6.3 nm and 22.0 ± 6.1 nm, respectively (Fig. S2). Their hydrodynamic sizes in the OECD (Organization for Economic Co-operation and Development) medium were determined to be 21.2 ± 1.4 nm and 33.9 ± 7.1 nm, respectively, largely comparable (*p* > 0.05) with their respective particle sizes, indicating the well dispersive mononanoparticles. The relatively larger hydrodynamic size than the particle size for P-AgNPs could be caused by the coated macromolecular PVP. Their measured zeta potentials were −43.8 ± 0.93 and −29.0 ± 0.58 mV, respectively, which provided strong electrostatic repulsions among NPs and thereby stabilized the NP suspensions in the OECD medium for a long time (>6 months). The steric repulsion originated from the PVP coating could also contribute to the stabilization of P-AgNPs.

### Characteristics of EPS and the EPS-extracted algae

**Figure 1:**
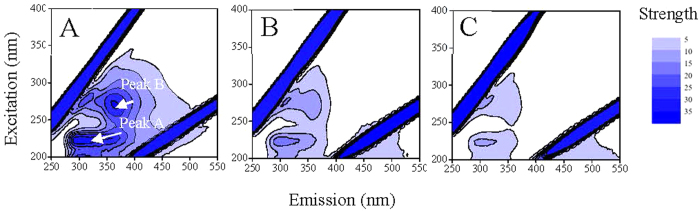
Three-dimension excitation (220–400 nm) and emission (300–550 nm) matrix fluorescence (3D-EEM) spectra of 25 mg/L EPS without (**A**) or with 25 mg/L C-AgNPs (**B**) or P-AgNPs (**C**), with the two fluorescence peaks indicated at EX/EM of 225/306 nm (peak A) and 275/360 nm (peak B). The 3D-EEM spectra of 25 mg/L EPS with other concentrations of AgNPs are shown in Fig. S5.

**Figure 2:**
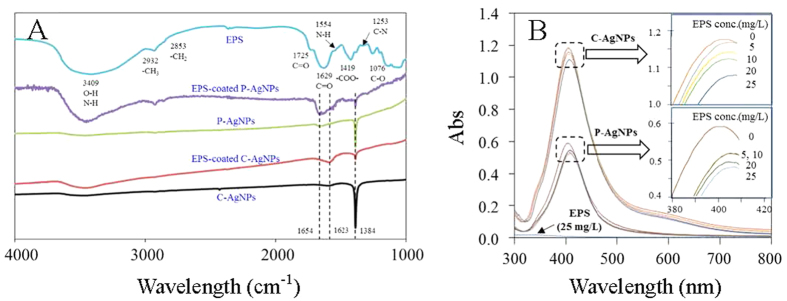
(**A**) Fourier transform infrared spectra of EPS, AgNPs, and the EPS-coated AgNPs; (**B**) UV-vis absorption spectra of 10 mg/L AgNPs with different concentrations of EPS. The two figures in part B are accordingly magnified from the two squared zones.

The extractable EPS was about 100 mg/g dry weight cells, comparable to the recorded 70.3 mg EPS/g *Rhodopseudomonas acidophila*^24^. Contents of total organic carbon (TOC), polysaccharide, and protein of the extracted EPS were 658, 684, and 270 mg/g, respectively, indicating the extracted EPS were mainly composed of polysaccharides and proteins. The three-dimension excitation (220–400 nm) and emission (300–550 nm) matrix fluorescence (3D-EEM) spectrum of EPS (Fig. 1A) shows two aromatic protein-like fluorescence peaks at EX/EM of 225/306 nm and 270/364 nm, evidencing the presence of protein in the EPS^25^. Fourier transform infrared (FTIR) spectrum of the extracted EPS is shown in Fig. 2A. The broad band at 3409 cm^−1^ was corresponded to -OH of carbohydrates and/or -NH of proteins; the bands at 2932 and 2853 cm^−1^ were related to the -CH_3_ and -CH_2_ stretching vibrations of fatty acid, respectively; the band at 1725 cm^−1^ was attributed to C=O stretching vibration of aromatic carboxylic acids; the three Amide absorption bands at 1629, 1554, and 1253 cm^−1^ were formed by the C=O stretching, N-H bending, and C-N stretching vibrations in the amino acids of proteins, respectively; the band at 1076 cm^−1^ was attributed to C-O stretching vibration in sugar derivatives, which together with the peak at 1725 cm^−1^ indicates the presence of a high amount of carboxylic acid^26^,^27^,^28^,^29^,^30^,^31^. These results suggest that the EPS mainly contained proteins and saccharides, and could have a good binding potential for heavy metals due to the abundant complexation functional groups like amido, carboxyl, and hydroxyl^32^,^33^.

Figure S3A,B show transmission electron microscope (TEM) images of the algal cells before and after the EPS extraction, respectively. The EPS-extracted algal cells remained intact in morphology, but had much thinner EPS layer dyed by the polysaccharide stain uranyl acetate. The Live/Dead test (Fig. S3C,D) and 96 h growth assay (Fig. S4A) show the EPS-extracted algae were active and propagated similarly as the intact algae with EPS. The kinetics of extracellular protein secretion of the algae with and without EPS is shown in Fig. S4B. During the 24 h incubation, the concentration of extracellular protein excreted from the intact and EPS-extracted algae (10^6^ cell/mL) increased from 1.17 mg/L to 1.58 mg/L and from 0.16 mg/L to 0.574 mg/L, respectively. The significantly (*p* < 0.05) lower concentration of extracellular protein excreted from the EPS-extracted algae suggested the EPS extraction was effective.

### Interactions between AgNPs and EPS

Figure 2B shows the effect of EPS on UV absorbances of AgNPs. C-AgNPs and P-AgNPs had similar UV absorbance spectra with a typical UV absorbance peak at 404 nm. Similar UV absorbance characteristics of AgNP suspensions have been reported elsewhere^34^. The UV absorbance of EPS was negligible, while the presence of EPS (5–25 mg/L) resulted in a decrease in strength and red shift of the UV absorbance peak of both AgNPs, indicating the interaction occurred between the AgNPs and EPS. The EPS adsorption could lower electronegativity and free electron density of the AgNPs, leading to a lower plasmon frequency (higher wavelength in UV spectrum)^35^,^36^,^37^. With 25 mg/L EPS, the peaks of C-AgNPs and P-AgNPs decreased in strength by 4.43% and 11.4% and shifted to 407 and 408 nm, respectively, indicating the EPS had a greater influence on the UV absorbance of P-AgNPs.

C-AgNPs and P-AgNPs had no fluorescence peaks, while they obviously affected the 3D-EEM spectrum of EPS (Fig. 1 and Table S1 in the SI). The two fluorescence peaks of EPS at EX/EM of 225/306 nm (peak A) and 275/360 nm (peak B) were lowered and shifted after the addition of 5–25 mg/L AgNPs, indicating the interaction occurred between AgNPs and proteins of EPS. The EPS-NP interaction could induce a static fluorescence quenching process, leading to the position shift (towards the lower emission wavelengths) and strength reduction of the peaks^38^. P-AgNPs had a greater effect on the 3D-EEM spectrum of EPS than C-AgNPs. In the presence of 25 mg/L C-AgNPs and P-AgNPs, the peak A of 25 mg/L EPS was lowered by 46.9% and 66.0% and shifted to 225/304 and 270/354 nm, and the peak B was lowered by 53.9% and 60.5% and shifted to 270/354 and 275/308 nm, respectively.

The EPS-NP interaction was further evidenced by the depression of typical FTIR peaks of AgNPs and the emergence of EPS FTIR peaks on the EPS-coated AgNPs (Fig. 2A). AgNPs themselves had no FTIR absorption band, and the FTIR peaks of C-AgNPs and P-AgNPs were derived from the coatings^39^,^40^. C-AgNPs had FTIR peaks at 1623 and 1384 cm^−1^, which were respectively attributed to C=O and C-O stretching vibrations of carboxyl in citrate; P-AgNPs had FTIR peaks at 1654 cm^−1^ (C=O stretching vibrations) and 1384 cm^−1^ (semi-circle ring stretching vibrations typically mixed with the C-H rock vibrations), which were derived from the pyrroles in PVP. After being coated by the EPS, the typical FTIR peak of C-AgNPs and P-AgNPs at 1384 cm^−1^ lowered, suggesting the EPS could more or less replace the surface coatings and bind to the AgNPs directly. Similar replace of surface coatings on NPs by organic macromolecules was also proposed by Diegoli *et al*.^41^ and Lau *et al*.^42^ The C=O stretching vibration peaks (at 1623 cm^−1^ for C-AgNPs and 1654 cm^−1^ for P-AgNPs) were however enhanced by the EPS coating owing to the abundant C=O groups in the EPS. The broad peak at 3409 cm^−1^ emerged on the EPS-coated P-AgNPs and C-AgNPs, verifying the surface coating of saccharides and/or proteins of EPS on the AgNPs. The peaks at 1076, 1253, 1554, and 1725 cm^−1^ in the EPS disappeared after coating on the NPs, implying the direct involvement of amino and aromatic carboxylic groups in the EPS-AgNP interaction. The greater alterations in the FTIR spectrum by the EPS-coating were observed for P-AgNPs than C-AgNPs, especially the presence of fatty acid peaks (2932 and 2853 cm^−1^) on the former but not on the later, suggesting P-AgNPs had a stronger interaction with EPS than C-AgNPs.

**Figure 3:**
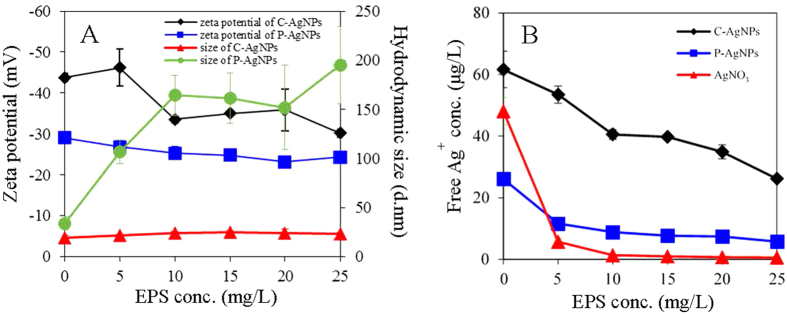
(**A**) Zeta potentials and hydrodynamic diameters of 10 mg/L AgNPs in the OECD medium with different concentrations of EPS; (**B**) free Ag^+^ (separated by 3 KDa Amicon centrifugal ultrafilter) concentrations of the OECD medium with 10 mg/L AgNPs or 50 μg/L Ag^+^ (AgNO_3_) in the presence of different concentrations of EPS.

The effects of EPS on zeta potential and hydrodynamic size of the two AgNPs are shown in Fig. 3A. Zeta potentials of C-AgNPs and P-AgNPs increased (*p* < 0.05) from −43.8 ± 0.93 and −29.0 ± 0.58 mV to −30.2 ± 0.94 and −24.3 ± 1.21 mV as the addition of EPS increased from 0 to 25 mg/L, respectively. The hydrodynamic size of P-AgNPs sharply increased (*p* < 0.05) and reached 195 ± 39 nm at 25 mg/L EPS, whereas the hydrodynamic size of C-AgNPs remained largely unchanged (*p* > 0.05). The greater interaction between EPS and P-AgNPs led to more amount of EPS adsorbed on P-AgNPs, and the adsorbed EPS could act as a bridge enhancing the NP aggregation, which could cause the larger aggregate size of P-AgNPs than C-AgNPs in the presence of EPS. The TEM observation (Fig. S1D) evidenced the presence of large NP aggregates in the P-AgNP suspension with EPS.

The effects of EPS on the free Ag^+^ concentrations in the test medium with the AgNPs or AgNO_3_ are shown in Fig. 3B. The AgNPs were partly soluble in the OECD medium. Ten mg/L C-AgNPs and P-AgNPs released 61.6 ± 5.9 μg/L and 26.1 ± 1.2 μg/L Ag^+^ in 24 h, respectively, indicating the higher solubility of C-AgNPs than P-AgNPs. The free Ag^+^ concentrations in the AgNP suspensions gradually decreased with increasing concentration of EPS (5–25 mg/L). In the presence of 25 mg/L EPS, the free Ag^+^ concentrations in the C-AgNP and P-AgNP suspensions were lowered to 26.1 ± 0.3 μg/L and 5.78 ± 0.86 μg/L, respectively. Extracellular proteins have been reported to chelate the released Ag^+^ from AgNPs and significantly reduce the free Ag^+^ concentration in the AgNP suspensions^32^,^33^,^43^,^44^. The free Ag^+^ concentration in the AgNO_3_ dissolution (50 μg Ag/L) was sharply lowered to 5.70 ± 1.11 μg/L at 5 mg/L EPS and nearly to zero at the higher concentrations, which evidenced the strong complexation of EPS with the dissolved Ag^+^ from the AgNPs. The AgNPs could continuously release dissolved Ag^+^ into the medium, making the final free Ag^+^ concentrations in the AgNP suspensions higher than that in the AgNO_3_ solution.

### The effect of EPS on the algal accumulations of AgNPs and Ag^+^

**Figure 4:**
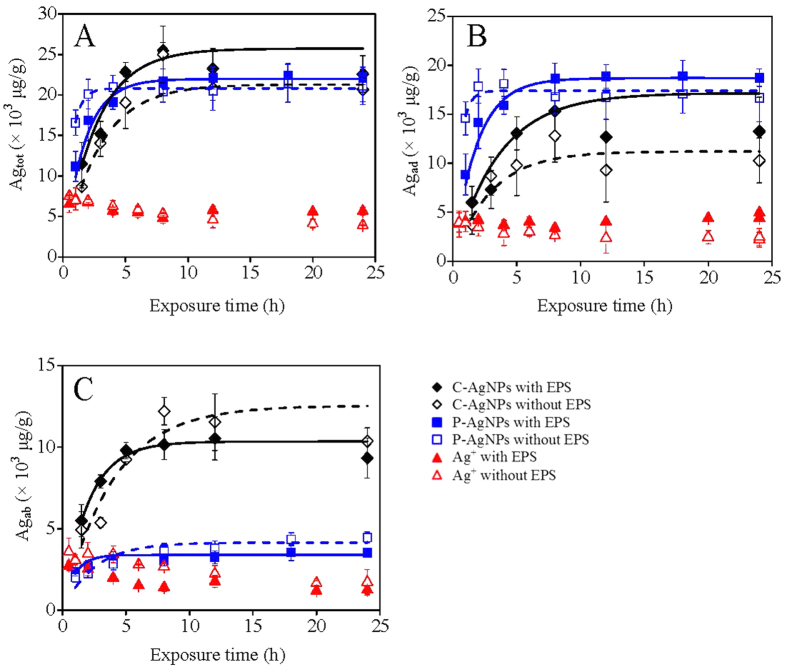
The kinetic total bioaccumulation (**A**), surface adsorption (**B**), and cell internalization (**C**) of AgNPs (50 μg/L) and Ag^+^ (10 μg/L) to the algae with or without EPS. The lines in the figure were the fitted results using the kinetic equation.

The kinetic accumulations of AgNPs and Ag^+^ to the algae are shown in Fig. 4, with the fitted rate constants for the total bioaccumulation (*k*_tot_), surface adsorption (*k*_ad_), and cell internalization (*k*_ab_) given in Table 1. It can be told that C-AgNPs had lower *k*_ad_ but much higher *k*_ab_ than P-AgNPs (*p* < 0.05). After the removal of EPS, *k*_tot_ and *k*_ad_ decreased but *k*_ab_ increased for both C-AgNPs and P-AgNPs (*p* < 0.05). The bioaccumulation of Ag^+^ was very much faster than that of the AgNPs, with all the three rate indexes reached equilibrium in half an hour. The EPS had no obvious effect on *k*_tot_ for Ag^+^ (10 μg/L), while the removal of EPS obviously lowered *k*_ad_ but increased *k*_ab_.

**Figure 5:**
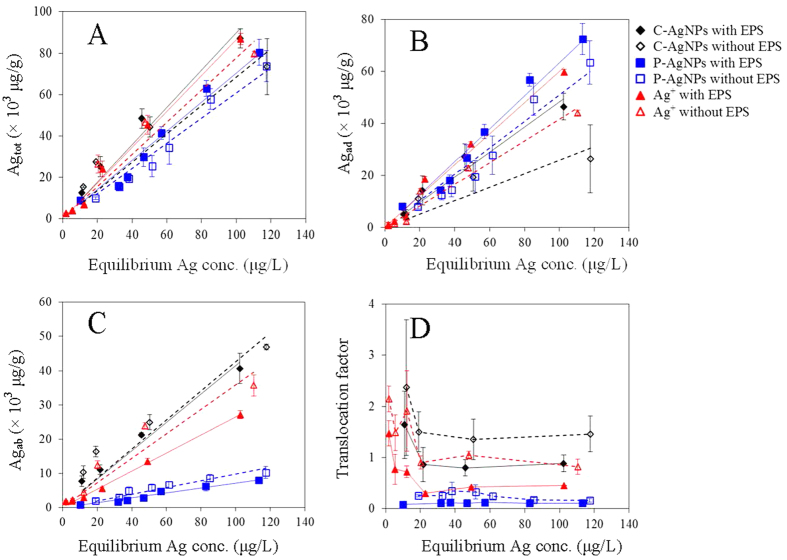
Variations of (**A**) the total bioaccumulation (Ag_tot_), (**B**) surface adsorption (Ag_ad_), (**C**) cell internalization (Ag_ab_) and (**D**) translocation factor of the AgNPs and Ag^+^ with the equilibrium Ag concentrations. The lines in figures (**A**–**C**) were the fitting results with the linear equation.

The thermodynamic accumulations of AgNPs and Ag^+^ to the algae are shown in Fig. 5. The total bioaccumulated (Ag_tot_), surface adsorbed (Ag_ad_), and cell absorbed (Ag_ab_) Ag all increased linearly (R^2^ = 0.948–0.996) with the addition of AgNPs or Ag^+^ within the text concentration ranges, with the fitted BCFs listed in Table 1. The total bioaccumulation factor (BCF_tot_) of the three Ag species ranged from 704 to 898 L/g, with an order of C-AgNPs > Ag^+^ > P-AgNPs. Yooiam *et al*.^45^ reported BCF_tot_ of uncoated AgNPs was 37.04 L/g to *Chlorella sp*. The BCF_tot_ in our study was much higher, which could be due to the higher stability of C-AgNPs and P-AgNPs than the naked AgNPs. The bioaccumulation could be substantially inhibited by the aggregation of naked AgNPs.

**Figure 6:**
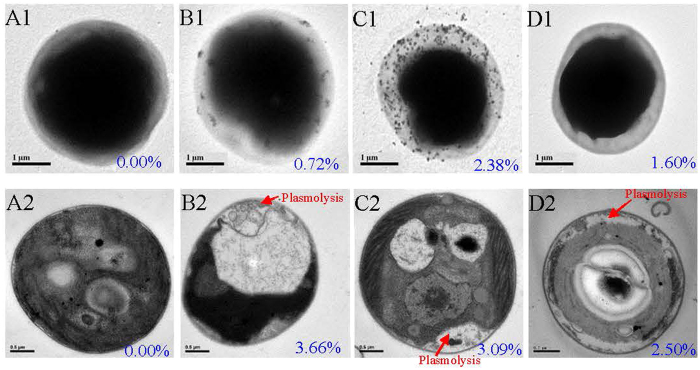
Transmission electron microscope images and X-ray energy dispersion spectroscopy determined Ag contents (the blue numbers in the images) of the intact (A1, B1, C1, and D1) and sectioned (A2, B2, C2, and D2) algal cells untreated (A1 and A2) or treated with 50 μg/L C-AgNPs (B1 and B2), 50 μg/L P-AgNPs (C1 and C2), or 10 μg/L Ag^+^ (D1 and D2).

The bioaccumulation factors based on surface adsorptions (BCF_ad_) of C-AgNPs, P-AgNPs, and Ag^+^ were 484 ± 32, 634 ± 21, and 601 ± 23 L/g, which accounted for about 53.9, 90.0, and 69.4% of their BCF_tot_, respectively; while the bioaccumulation factors based on cell absorptions (BCF_ab_) of the three Ag species were 415 ± 21, 70.7 ± 2.7, and 265 ± 4 L/g, respectively. Compared with Ag^+^, C-AgNPs had comparable BCF_tot_ (*p* > 0.05) but much higher BCF_ab_ (*p* < 0.05). P-AgNPs had the smallest total bioaccumulation (*p* < 0.05) and were mainly adsorbed on the algal surfaces. The translocation factors (TFs) are shown in Fig. 5D. The TF of C-AgNPs was higher than P-AgNPs and Ag^+^. This could be due to the highly negative charge of C-AgNPs that limited the electrostatic attraction to the cell surfaces and the small size of C-AgNPs that facilitated the cell internalization. The bioaccumulation and distribution of the three Ag species were further analyzed by the TEM and X-ray energy dispersion spectroscopy (EDS) (Fig. 6). Much more amount of NPs was observed on the surfaces of intact algal cells with P-AgNPs than C-AgNPs, indicating the stronger interaction between P-AgNPs and EPS. AgNPs could be internalized into the cell interior^4^, but no NPs were observed in the sectioned cells, which could be due to the small size of internalized dispersed-mononanopartilces and/or the dissolution of AgNPs in the cells. The EDS measurements confirmed the bioaccumulation of Ag to the algal cells. Ag was detected on and in the treated algal cells but not the control cells. After the treatment with the same Ag concentration, the order of EDS-determined Ag contents on the surfaces of intact cells was P-AgNP > AgNO_3_ > C-AgNPs, the same as the order of their BCF_ad_. However for the sectioned cells, the highest Ag content was detected for the treatment of C-AgNPs, suggesting the algal cells had higher absorption though lower adsorption for C-AgNPs than P-AgNPs. This is consistent with the result of thermodynamic bioaccumulation experiment.

As shown in Fig. 5, when EPS was removed the order of bioaccumulations for the three Ag species kept largely unchanged, whereas Ag_tot_ and Ag_ad_ decreased and Ag_ab_ increased. Consequently, TFs of the three Ag species all increased after the EPS removal. EPS could act as the first bio-barrier outside the cells to complex with Ag ions and NPs, thus the cells with EPS had higher BCF_ad_. However the EPS-Ag complex could be stable and hard to be absorbed into the cells^46^,^47^. When EPS was removed, there were more chances for Ag ions and NPs to contact with the cytomembrane directly and be internalized; therefore although BCF_ad_ decreased after the removal of EPS, BCF_ab_ and TF increased.

After the removal of EPS, BCF_ad_ of C-AgNPs, P-AgNPs, and Ag^+^ decreased (*p* < 0.05) by about 46.5%, 19.5%, and 30.6%, while their BCF_ab_ increased by about 2.89%, 27.3% (*p* < 0.05), and 26.2% (*p* < 0.05), respectively. P-AgNPs had the lowest BCF_ad_ reduction, implying P-AgNPs could strongly bind with both the EPS and the EPS-extracted cell surfaces. C-AgNPs had the lowest BCF_ab_ increase, indicating the EPS had a limited effect on the cell internalization of C-AgNPs. After the removal of EPS, the increases in TF of C-AgNPs and Ag^+^ were comparable, which were significantly higher than that of P-AgNPs. But the reasons for the TF increases of C-AgNPs and Ag^+^ were different. The EPS removal markedly decreased the surface adsorption and slightly increased the cell internalization of C-AgNPs, while it almost equally decreased the surface adsorption and increased the cell internalization of Ag^+^.

The uptake of AgNPs by microbes has been studied^33^,^48^,^49^,^50^, while the contributions of NPs and their released Ag^+^ in the bioaccumulation remain debatable. It is recognized that algae can absorb the released Ag^+^ from AgNPs. However, the limited dissolution (<10%) of the AgNPs and the largely comparable BCF_tot_ between the AgNPs and Ag^+^ (704–898 L/g) suggested that the algae could accumulate the AgNPs directly besides the released Ag^+^. The cell surfaces are generally heterogeneous in terms of binding affinity. Ag at low concentrations would be preferably attracted to surface sites with high binding affinity and then occupy the weaker binding sites with increasing Ag concentration and after the saturation of the strong binding sites. Surface washing using cysteine may be unable to desorb a fraction of the strongly-bound Ag on the cell surfaces, which could more or less account for the apparently higher TFs at the low Ag concentrations especially of C-AgNPs and AgNO_3_ (Fig. 5D).

### The effect of EPS on the algal toxicity of AgNPs and Ag^+^

**Figure 7:**
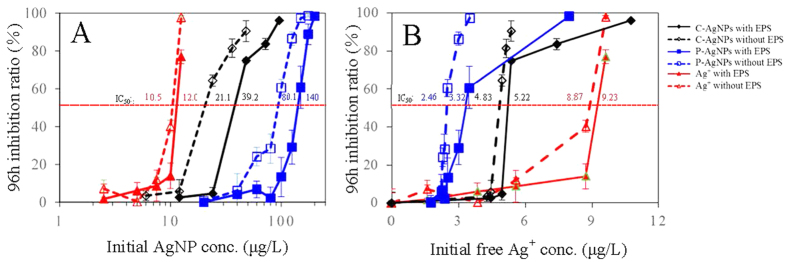
The 96 h dose-response curves of the AgNPs and Ag^+^ based on the concentrations of (**A**) total Ag and (**B**) free Ag^+^ ions in the exposure media to the algae.

The dose-response curves of AgNPs and Ag^+^ on the 96 h algal growth are shown in Fig. 7A. The 96 h 50% algal cell growth inhibition concentrations (IC_50_) of Ag^+^, C-AgNPs, and P-AgNPs to the intact algae with EPS were 12.0, 39.2, and 140 μg/L, respectively. As shown in the TEM images of the sectioned cells (Fig. 6), the control cell was intact in morphology, while plasmolysis and membranolysis occurred to the Ag-treated cells, evidencing the algal toxicity of the AgNPs and Ag^+^. At the same Ag concentration, the plasmolysis and membranolysis of algal cells exposed to Ag^+^ were apparently more serious than the ones exposed to C-AgNPs and P-AgNPs, verifying the toxicity order of Ag^+^ > C-AgNPs > P-AgNPs. Brad *et al*.^51^ also studied the toxicity of the three Ag species to the fresh water algae *Pseudokirchneriella subcapitata* and reported 72 h IC_50_ of 1.1, 3.0, and 19.5 μg/L, respectively, which were about one order higher in toxicity but the same in the toxicity sequence compared with our results. The different toxicity could be due to the different sensitivity to Ag of the two algal species. The 96 h IC_50_ of the three Ag species to the EPS-extracted algae were lowered to 10.5, 21.1, and 80.1 μg/L, respectively, suggesting the mitigation effect of EPS on the toxicity.

Figure 7B shows the dose-response curves of algae to the measured free Ag^+^ in the tested NP suspensions and AgNO_3_ solutions. In this case, the toxicity order of the three Ag species was reversed, and IC_50_ of C-AgNPs and P-AgNPs were 5.22 and 3.32 μg Ag^+^ /L, respectively, which were lower than IC_10_ of Ag^+^, suggesting the observed nanotoxicity was not from the initially released Ag^+^ in the AgNP suspensions. The AgNPs during the toxicity test could further release dissolved Ag^+^ into the culture medium with the cell accumulation of Ag^+^, contributing to the observed toxicity. But the cell surface adsorbed and/or internalized AgNPs may play a role in the nanotoxicity. P-AgNPs had higher surface adsorption but much lower toxicity than Ag^+^, suggesting the surface adsorbed AgNPs had limited contribution to the observed nanotoxicity. The cell-absorbed AgNPs and/or Ag^+^ may be further transformed in the intracellular system, inducing a series of toxic effects like oxidative stress, impairment of the cell membrane, DNA damage, genotoxicity, and so on^52^,^53^,^54^. But whether and how AgNPs and/or Ag^+^ play these toxic roles are still unclear, and the exact transformation and toxic mechanism of the cell-internalized AgNPs and/or Ag^+^ warrant more studies.

The above results, i.e., the increasing production of EPS induced by the AgNPs/Ag^+^, the EPS-AgNPs/Ag^+^ interaction, the inhibition of EPS on the cell internalization of AgNPs/Ag^+^, and the alleviation effect of EPS on the algal toxicity of AgNPs/Ag^+^, demonstrated that EPS could alleviate the algal toxicity of AgNPs not only by reducing the concentration of released free Ag^+^ in the culture medium^44^ and inhibiting the cell internalization of Ag^+^, but also by restraining the toxic pathway of “Trojan-horse” mechanism through limiting the internalization of AgNPs. Compared with P-AgNPs, C-AgNPs had lower algal adsorption but higher Ag^+^ dissolution and cell internalization, and were therefore more toxic to the algae.

## Methods

### Synthesis and characterization of AgNPs

C-AgNPs and P-AgNPs were synthesized from a standard reduction of the silver salt^55^,^56^, with the method detailed in the SI. Morphology of the AgNPs was examined with the TEM (JEM1230, Japan). Hydrodynamic size and zeta potential were characterized using a zetasizer (Nano ZS90, Malvern Instruments, UK). Concentration of the AgNP suspensions was measured by inductively coupled plasma mass spectrometry (ICP-MS) after the acid digestion.

### Extraction and characterization of EPS

The green alga *Chlorella pyrenoidosa*, having been used in our previous studies^57^,^58^, was cultured in the OECD medium. EPS were extracted from the algae by the cation exchange resin method^59^ (detailed in the SI) and stored at −20 °C. Carbonhydrate and protein contents of EPS were measured by the anthrone method^60^ and bicinchoninic acid assay^61^, respectively. The 3D-EEM spectrum of the extracted EPS was obtained with a spectrofluorometer (Hitachi F-4600, Japan). FTIR spectrum of EPS was recorded on an infrared spectroscope (Nicolet 6700, Thermo scientific, USA). The TOC content was measured by a TOC analyzer (Shimadzu TOC-VCPN analyzer, Kyoto, Japan).

After the EPS extraction, the remaining algae were collected and regarded as the algae without EPS. The TEM characterization, Live/Dead test^62^, and 96 h growth assay were performed to assess the potential effect of the EPS extraction on the algae. As one of the major components of EPS, the secretion kinetics of extracellular protein from the untreated and EPS-extracted algal cells in the absence and presence of the AgNPs and Ag^+^ (from AgNO_3_) were also determined. Details of the experimental designs are described in the SI.

### The interaction between EPS and AgNPs

Different doses of EPS (0–25 mg/L) were added into the AgNP suspensions (10 mg/L), and the mixtures were subsequently shaken (120 rpm) for 24 h in darkness. A fraction of the resultant suspensions was collected to measure the hydrodynamic size and zeta potential by the zetasizer for the analysis of the potential effect of EPS on the colloidal properties of AgNPs. An aliquot of the suspensions was measured by an UV-vis absorption spectroscope (Shimadzu UV2450, Kyoto, Japan). In the meantime, different doses of AgNPs (0–25 mg/L) were added into the EPS solution (25 mg/L). After the shaking at 120 rpm for 24 h in darkness, 3D-EEM spectra of the mixtures were measured to analyze the effect of AgNPs on the fluorescence characteristics of EPS. FTIR spectra of the AgNPs and the EPS-coated AgNPs were also measured to analyze the EPS-AgNP interactions, with the method detailed in the SI.

The released free Ag^+^ ions from the AgNPs in the presence of different concentrations of EPS were measured. Different doses of EPS (0–25 mg/L) were added into the AgNP suspensions (10 mg/L) and AgNO_3_ solution (50 μg Ag/L, which was comparable to the concentration of free Ag^+^ released by 10 mg/L AgNPs). After the shaking (120 rpm) for 24 h in darkness, a fraction of the mixtures was centrifuged at 8000 *g* for 30 min using Amicon centrifugal ultrafilter (3 KDa, Millipore, USA) to remove the AgNPs. The concentrations of free Ag^+^ in the obtained filtrates were determined by the ICP-MS.

### Bioaccumulation experiments

The effect of EPS on algal accumulations of AgNPs and Ag^+^ (from AgNO_3_) was examined by kinetic and thermodynamic bioaccumulation tests. The algae with or without EPS were exposed to the three Ag suspensions and cultured in an incubator (120 rpm) at 25 °C. Dissolved organic matter could more or less reduce Ag^+^ to metallic particles in the presence of light^63^, and sunlight was observed to induce the aggregation of polymer-stabilized AgNPs^64^. Therefore, the bioaccumulation experiments were operated in darkness. In the kinetic experiment, the concentrations of AgNPs and Ag^+^ were fixed at 50 and 10 μg/L, respectively, and various exposure times (0–24 h) were selected. At these concentrations the cell survival rates were above ninety percent according to a preliminary test. In the thermodynamic experiment, the exposure time was fixed at 8 h (enough to reach the bioaccumulation equilibrium according to the kinetic experiment) and different Ag concentrations were tested.

After the exposures, the algal cell density in the culture medium was measured using a counting chamber under a light microscope (LM, Olympus, CX21, Japan) and divided by the unit algal dry weight (which was measured by the gravimetric method) to obtain the algal biomass (g/L). The culture medium was then centrifuged at 2500 *g* for 10 min. The resultant supernatant free of algal cells was collected, digested, and measured by the ICP-MS to get the concentration of water column Ag (μg/L). The depositions of the C-AgNP and P-AgNP suspensions by the centrifugation were negligible according to a preliminary experiment, and therefore the total bioaccumulation (adsorption and absorption) of Ag (Ag_tot_, μg/g) was calculated by the division of the decrement between the initial and residual water column Ag concentrations by the algal biomass. The deposited algae by the centrifugation were collected and washed with 5 × 10^−3^ M cysteine twice in order to remove the potentially adsorbed Ag on the cell surfaces. The cysteine washing has been reported to efficiently remove the adsorbed Ag from organism surfaces including algae^65^,^66^,^67^. The washed algae were weighted and analyzed by the ICP-MS after the acid digestion. The measured cell-internalized Ag concentration was divided by the cell biomass to get the concentration of algae-absorbed Ag (Ag_ab_, μg/g). The cell-adsorbed Ag (Ag_ad_, μg/g) was then calculated by the decrement between Ag_tot_ and Ag_ab_. The Ag_ab_ to Ag_ad_ ratio was served as the TF, representing the translocation ability of Ag from algal surfaces to cell interior.

To quantitatively compare the bioaccumulation rates of the three Ag species, we fitted the kinetic bioaccumulation results by the kinetic equation (Eq. 1).


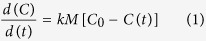


where *C*(t) (μg/g) was the bioaccumulated concentration of Ag (Ag_tot_, Ag_ad_ or Ag_ab_) at time *t*, *k* (μg^−1^Lh^−1^) was the bioaccumulation rate constant, M (μg/L) was the used Ag concentration in the culture medium, and *C*_0_ was the maximum bioaccumulated concentration.

The thermodynamic bioaccumulation results were fitted by the linear bioaccumulation equation (Eq. 2):


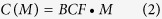


where *C*(M) (μg/g) was the concentration of total bioaccumulated, surface adsorbed or absorbed Ag (Ag_tot_, Ag_ad_ or Ag_ab_, respectively) at a given equilibrium Ag concentration in the culture medium (M, μg/L), and BCF (L/g) was the bioaccumulation factor based on the total bioaccumulation (BCF_tot_), surface adsorption (BCF_ad_) or absorption (BCF_ab_).

### Algal toxicity experiment

The growths of algal cells with and without EPS exposed to various concentrations of AgNPs and Ag^+^ (from AgNO_3_) were measured and compared to evaluate the impacts of EPS on the algal toxicity of Ag. The intact or EPS-extracted algal cells (1.5 × 10^5^ cells/mL) were cultured in a 250 mL Erlenmeyer flask containing 100 mL of the OECD medium with various concentrations of Ag. The flasks were kept on a rotary shaker (120 rpm) at 25 °C with illumination by white incandescent lights (70 μmol photons/m^2^/s, light: dark of 14:10 h). After the exposure for 96 h, the algal cells in the medium were counted. Dose-response curves of the two AgNPs and Ag^+^ ions towards the algal cells with or without EPS were obtained and IC_50_ were calculated using the linear interpolation method. The free Ag^+^ in each concentration of AgNPs and AgNO_3_ solutions were measured by the ICP-MS after the ultrafiltration to evaluate the potential contribution of released Ag^+^ ions to the apparent nanotoxicity.

### Electron microscopy and EDS characterizations

A drop of the algal suspension after the exposure to the three Ag suspensions (50 μg/L for the AgNPs, 10 μg/L for Ag^+^) for 8 h was air-dried onto a copper grid and the morphology of algal cells was observed with the TEM. To observe potential changes of the cellular structure, the treated and untreated algal cells were fixed in 2.5% glutaraldehyde, dehydrated by ethanol and acetone, embedded, stained with OsO_4_, sectioned, counterstained, and then observed by the TEM with the same method applied in our previous studies^57^,^68^,^69^. Both the intact cells and sectioned cells were analyzed by the EDS (GENESIS 4000, EDAX Inc., USA) to determine the Ag contents on and in the cells, respectively^70^,^45^.

### Statistical analysis

All of the experiments were run in triplicate and the data were presented as mean ± standard deviations. The differences in the bioaccumulation parameters between different treatments were analyzed by the *t*-test with Medcalc Software, while the differences between the control and the treatments in the other experiments were analyzed by ANOVA with SPSS, with *p* ≤ 0.05 defined as significant.

## Additional Information

**How to cite this article**: Zhou, K. *et al*. The role of exopolymeric substances in the bioaccumulation and toxicity of Ag nanoparticles to algae. *Sci. Rep.* **6**, 32998; doi: 10.1038/srep32998 (2016).

## Supplementary Material

Supplementary Information

## Figures and Tables

**Table 1:** The fitted parameters for the kinetic and thermodynamic bioaccumulations of the AgNPs and Ag^+^ to the algae.

		the kinetic bioaccumulation						the thermodynamic bioaccumulation					
Ag species		*k*_tot_	R^2^_tot_	*k*_ad_	R^2^_ad_	*k*_ab_	R^2^_ab_	BCF_tot_	R^2^_tot_	BCF_ad_	R^2^_ad_	BCF_ab_	R^2^_ab_
C-AgNPs	With EPS	0.515 ± 0.031	0.830	0.344 ± 0.027	0.907	0.206 ± 0.007	0.938	898 ± 49	0.962	484 ± 32	0.948	415 ± 21	0.966
	Without EPS	0.426 ± 0.017	0.982	0.224 ± 0.027	0.845	0.250 ± 0.040	0.770	685 ± 80	0.785	259 ± 39	0.626	427 ± 43	0.848
P-AgNPs	With EPS	0.440 ± 0.008	0.937	0.374 ± 0.006	0.942	0.068 ± 0.019	0.798	704 ± 23	0.972	634 ± 21	0.972	70.7 ± 2.7	0.966
	Without EPS	0.417 ± 0.003	0.960	0.348 ± 0.006	0.661	0.083 ± 0.005	0.858	607 ± 23	0.963	510 ± 26	0.942	97.3 ± 3.6	0.925
Ag^+^	With EPS							865 ± 23	0.991	601 ± 23	0.983	265 ± 4	0.996
	Without EPS							775 ± 52	0.946	417 ± 23	0.964	359 ± 30	0.905

Note: *k* was the overall rate constant for the bioaccumulations (μg^−1^Lh^−1^), BCF was the bioaccumulation factor (L/g), and R^2^ was the goodness of the fitting. The bioaccumulation of Ag^+^ was too fast to be fitted by the kinetic equation in this study.
