# Supplementary material for: The role of exopolymeric substances in the bioaccumulation and toxicity of Ag nanoparticles to algae
Source: Sci Rep. 2016 Sep 12;6:32998. doi: 10.1038/srep32998 (PMC5018811; doi:10.1038/srep32998)
Supplement: Supplementary Information [file srep32998-s1.pdf]

Supplementary information

The role of exopolymeric substances in the bioaccumulation and toxicity of Ag nanoparticles to algae

Kaijun Zhou[1], Yi Hu[1], Luqing Zhang[1], Kun Yang[1,2], Daohui Lin[1,2,*]

1. Department of Environmental Science, Zhejiang University, Hangzhou 310058, China

2. Zhejiang Provincial Key Laboratory of Organic Pollution Process and Control, Zhejiang University, Hangzhou 310058, China

*Corresponding author: Tel.: +86 571 88982582; fax: +86 571 88982590.

E-mail address: lindaohui@zju.edu.cn (D. Lin)

Experimental Procedures

Synthesis of AgNPs. Citrate stabilized AgNPs (C-AgNPs) were synthesized according to the reported method[1] from a standard reduction of the silver salt in sodium citrate. One hundred mL solutions of sodium citrate (0.31 mM), $AgNO_3$ (0.25 mM) and sodium borohydride ($NaBH_4$, 0.25 mM) were prepared in pure water and kept at 4 °C in darkness for 30 min. The $AgNO_3$ and sodium citrate solutions were mixed together in a conical flask and vigorously stirred. Six mL of the $NaBH_4$ solution was added into the mixture. After stirring for 10 min, the solution was heated slowly to boiling for 90 min, left overnight and then cooled (4 °C, in darkness), and collected as the C-AgNP suspension. While polyvinyl pyrrolidone (PVP) stabilized AgNPs (P-AgNPs) were synthesized according the reported method[2] from the reduction of the silver salt in PVP_{10}. One hundred mL solutions of PVP_{10} (2 mM), $AgNO_3$ (1 mM) and $NaBH_4$ (4 mM) were prepared in pure water and kept at 4 °C in darkness for 30 min. The PVP_{10} and $NaBH_4$ solutions were mixed together and vigorously stirred. 70 mL of the $AgNO_3$ solution was added into the mixture, then the stirring was stopped simultaneously, and the suspension was left overnight and collected as the P-AgNP suspension. Both the obtained C-AgNP and P-AgNP suspensions were cleaned at least three times using the ultrafiltration method (Ultrafiltration centrifuge tube, 3 KDa, Millipore, USA) to remove the excessive reactants, preventing aggregation and drying. The cleaned AgNP suspensions were kept in darkness at 4 ºC.

EPS extraction. EPS were extracted by the cation exchange resin method[3]. *Chlorella pyrenoidosa* was cultivated to the stationary phase and was harvested by the centrifugation at 2500*g*. After being washed twice by the culture medium, the algae were mixed with 2.5 g/(biomass) g cation exchange resin (Dowex Marathon C) and stirred at 600 rpm for 1.5 h. Then the mixture was centrifuged at 12000*g* for 15 min. The supernatant rich of EPS was desalinized, concentrated by an ultrafiltration system (Sartorius Vivaflow200, Germany), and freeze-dried to get the EPS solid. The harvested EPS were stored at -20 ºC before using.

TEM observation, Live/Dead test and growth assay of algae with or without EPS. After the EPS extraction, the remaining algae were collected and regarded as the algae without EPS. The transmission electron microscope (TEM) characterization, Live/Dead test[4], and 96h growth assay were performed to assess the potential effect of the EPS extraction on the algae. For the TEM observation, algae with or without EPS were dyed by the polysaccharide stain uranyl acetate. A drop of algal suspension was air-dried onto a copper grid and was observed by using a TEM

(JEM-1230, Japan). The Live/Dead Baclight Bacterial Viability Kit (Invitrogen/Molecular Probes, USA) was used to distinguish live and dead cells, which utilized mixtures of green-fluorescent nucleic acid stain (SYTO 9) and red-fluorescent nucleic acid stain (propidium iodide, PI). The SYTO 9 stain generally labels all cells when used alone, while PI can only penetrate cells with damaged membranes, causing a reduction in the SYTO 9 stain fluorescence when both dyes are present. The stained cells were imaged with a fluorescence microscope (FM, Nikon Eclipse Ni, Japan), and the live and dead cells were distinguished. For the 96h growth assay, algae (1.5×10^5 cells/mL) were cultured in a 250 mL Erlenmeyer flask containing 100 mL of the OECD medium. The flasks were kept on a rotary shaker (120 rpm) at 25 ºC with illumination by white incandescent lights (70 μmol photons/m^2/s, light:dark of 14:10h). After being cultivated for different times (12, 24, 48, 72, 96, and 120 h), the algal cells in the media were counted using a counting chamber under a light microscope (LM, Olympus, CX21, Japan).

The secretion kinetics of extracellular protein. As one of the major components of EPS, extracellular protein was measured to evaluate the EPS content. The intact algae or algae with EPS extracted (10^6 cells/mL) were exposed in the OECD medium with or without Ag (50 μg/L AgNPs or 10 μg/L $AgNO_3$), and cultured in an incubator (120 rpm) at 25 ºC in darkness. Then the algal cells were collected by centrifugation at 2500*g* for 10 min after the exposure for different times. The EPS on the cell surfaces were extracted by the cation exchange resin method as above. The concentration of extracellular protein in the EPS was measured by the bicinchoninic acid assay[5].

The effect of EPS on FTIR spectra of AgNPs. The EPS solution (250 mg/L) was mixed with the AgNP suspensions (100 mg/L), shaking for 24 h at 25 ºC in darkness. The stock AgNP suspensions and the EPS-AgNP mixtures were both centrifuged at 15000*g* for 30 min to collect the AgNP and EPS-coated AgNP precipitates, respectively. The collected precipitates were washed twice with ultrapure water and freeze dried. The FTIR spectra of 1 mg of EPS, AgNPs or EPS-coated AgNPs mixed with 99 mg of KBr powder were obtained on an infrared spectroscope (Nicolet 6700, Thermo scientific, USA).

References

1. Romer, I. et al. Aggregation and dispersion of silver nanoparticles in exposure media for aquatic toxicity tests. *J. Chromatogr. A* **1218,** 4226–4233 (2011).

2. Mulfinger, L. et al. Synthesis and study of silver nanoparticles. *J. Chem. Educ.* **84,** 322 (2007).

3. Long, G. Y., Zhu, P. T., Shen, Y. & Tong, M. P. Influence of extracellular polymeric substances (EPS) on deposition kinetics of bacteria. *Environ. Sci. Technol.* **43,** 2308–2314 (2009).

4. Ivanova, I. A. et al. Determination of pseudomonas putida live cells with classic cultivation and staining with “live/dead baclight bacterial viability kit”. *Biotechnol. Biotechnol. Equip.* **24,** 567–570 (2014).

5. Walker, J. M. The bicinchoninic acid (BCA) assay for protein quantitation. *Methods Mol. Biol.* **32,** 11–14 (1994).

Table S1. The effect of different concentrations of AgNPs on the position and strength of three-dimension excitation (220−400 nm) and emission (300−550 nm) matrix fluorescence peaks of 25 mg/L EPS.

Ag concen. (mg/L)	C-AgNPs				P-AgNPs			
	Peak A		Peak B		Peak A		Peak B	
	Ex/Em	strength	Ex/Em	strength	Ex/Em	strength	Ex/Em	strength
0	225/306	34.7	270/364	30.6	225/306	34.7	270/364	30.6
5	225/302	34.1	270/362	28.2	225/302	29.3	270/356	25.8
10	225/298	28.9	270/360	24.2	225/302	25.7	270/356	20.4
15	225/300	26.6	270/358	21.3	225/304	20.7	270/354	15.7
20	225/302	22.7	270/358	19.0	225/304	14.3	275/352	11.0
25	225/304	18.4	270/354	14.0	225/302	11.8	275/308	12.0

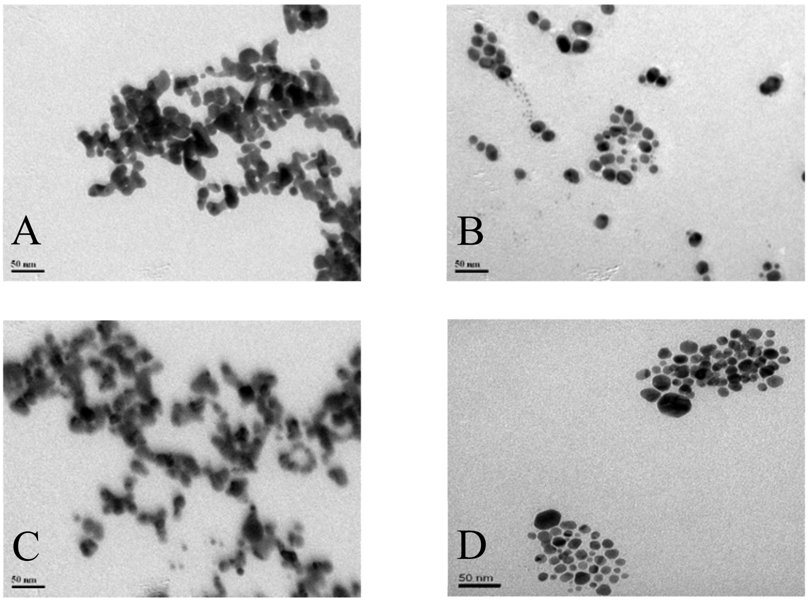

Fig S1. Transmission electron microscope images C-AgNPs (A and C) and P-AgNPs (B and D) in the absence (A and B) and presence (C and D) of 25 mg/L EPS.

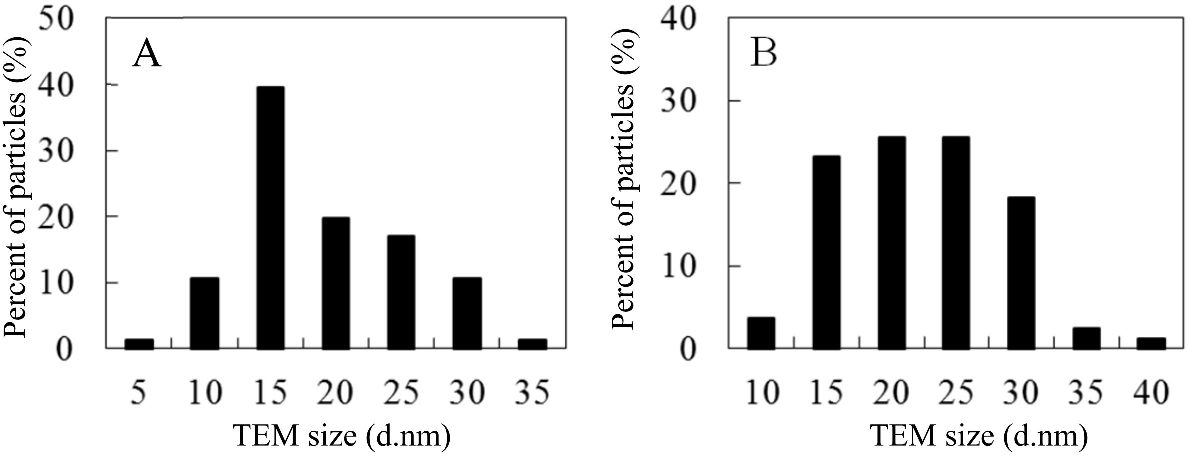

Fig S2. Size distribution of C-AgNPs (A) and P-AgNPs (B) measured for the particles (n>100) shown in the transmission electron microscope images.

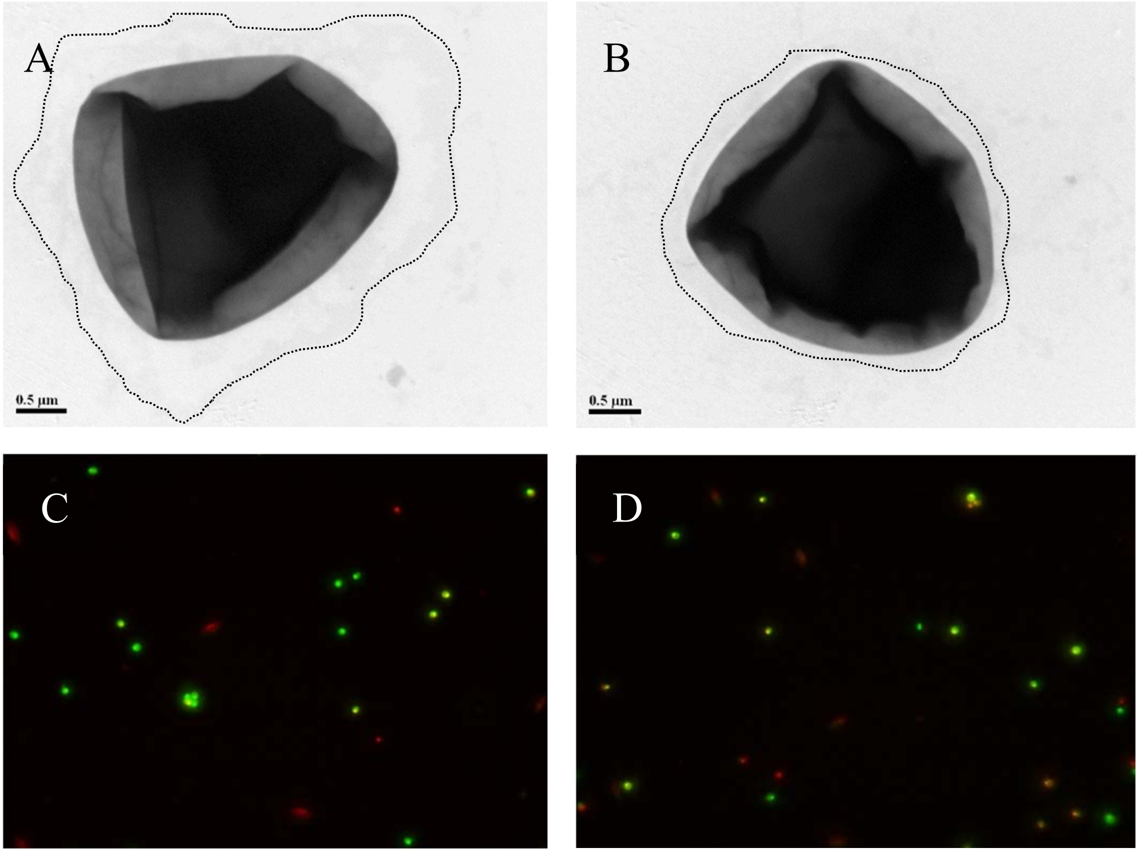

Fig S3. Transmission electron microscope images of the intact algal cells (A) and EPS-extracted cells (B), with the extracellular polysaccharide layers dyed by the polysaccharide stain uranyl acetate and circled by dashed lines; fluorescence microscope images of the intact algal cells (C) and EPS-extracted cells (D), with the live cells dyed by the green-fluorescent nucleic acid stain (SYTO 9) and the dead cells dyed by the red-fluorescent nucleic acid stain (propidium iodide, PI).

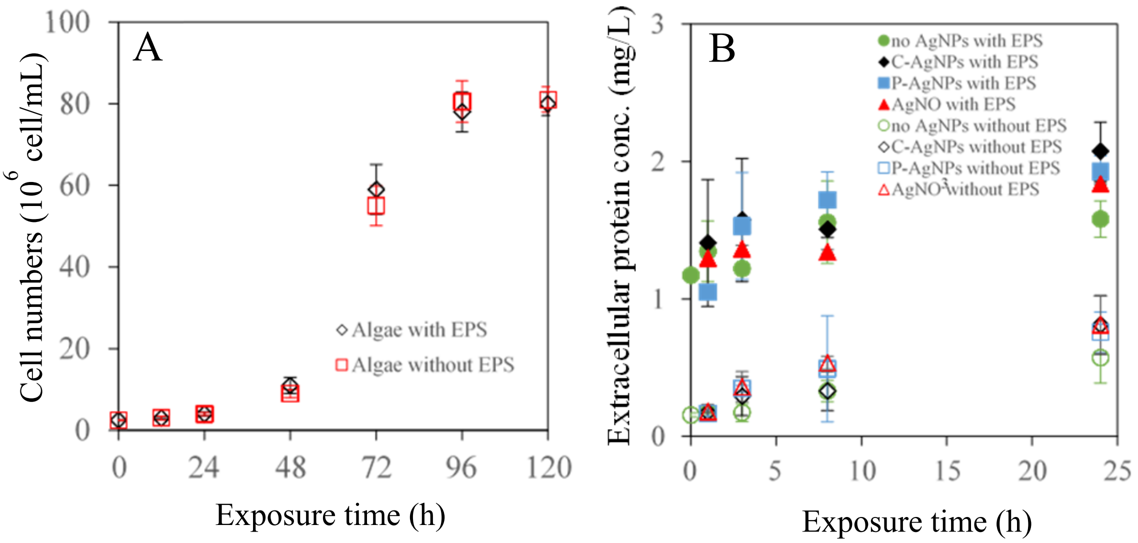

Fig S4. (A) Growth curves of algae with or without EPS and (B) secretion of extracellular protein by algae with or without EPS in the absence or presence of 50 μg/L AgNPs or 10 μg/L $AgNO_3$.

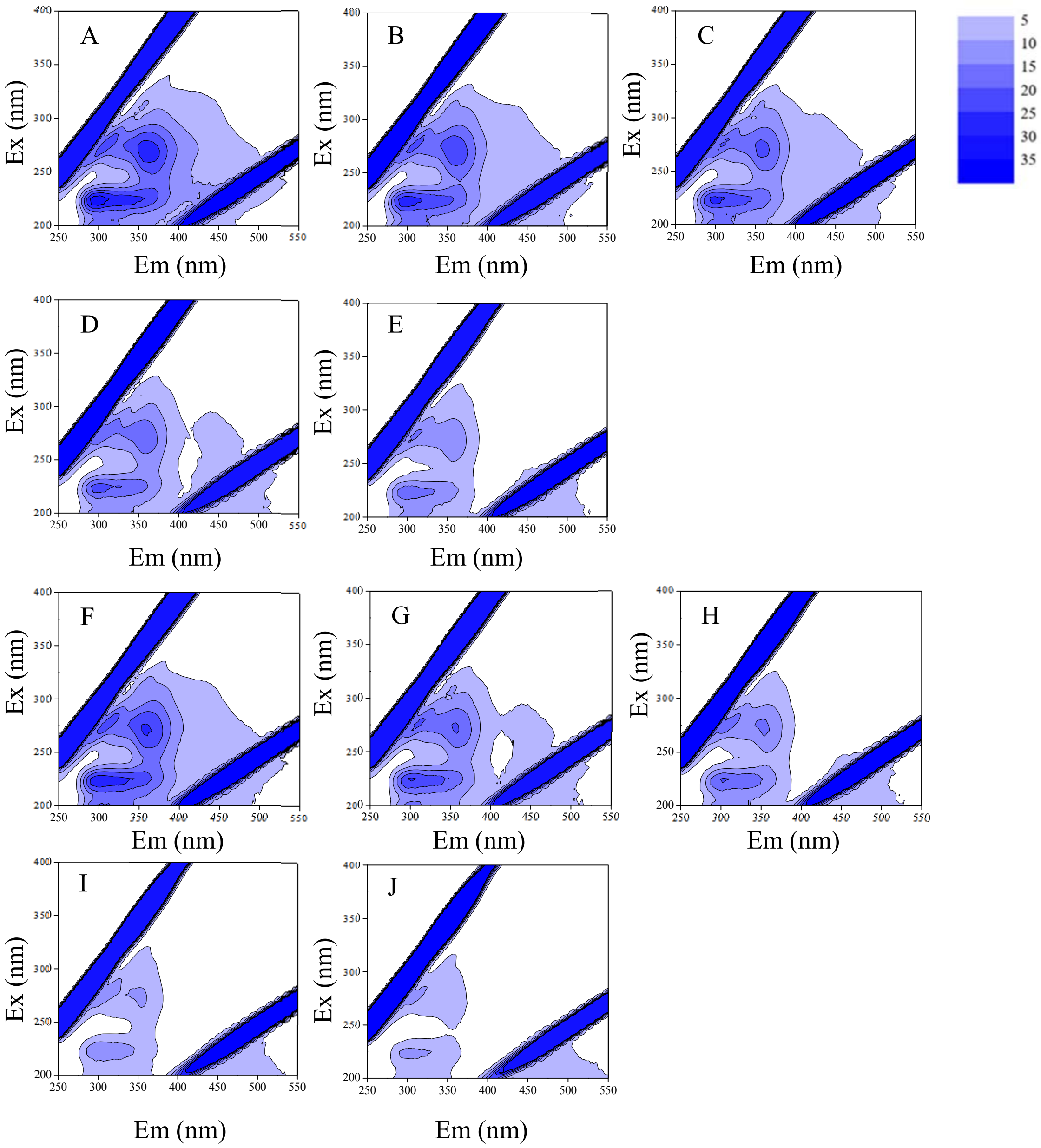

Fig. S5. Three-dimension excitation (220−400 nm) and emission (300−550 nm) matrix fluorescence (3D-EEM) spectra of 25 mg/L EPS with different concentrations (5, 10, 15, 20, and 25 mg/L) of the AgNPs (from A to E for C-AgNPs and from F to J for P-AgNPs, respectively), with two fluorescence peaks of EPS at EX/EM of 225/306 nm (peak A) and 275/360 nm (peak B) lowered and shifted after the addition of 5-25 mg/L AgNPs.
